# Differences in Biogeographic Patterns and Mechanisms of Assembly in Estuarine Bacterial and Protist Communities

**DOI:** 10.3390/microorganisms13010214

**Published:** 2025-01-20

**Authors:** Weiyue Zhang, Yunlei Zhang, Zhizhuo Shao, Yi Sun, Hongjun Li

**Affiliations:** State Environmental Protection Key Laboratory of Coastal Ecosystem, National Marine Environmental Monitoring Center, Dalian 116023, China; zwy2017506@163.com (W.Z.); ylzhang@nmemc.org.cn (Y.Z.); shao_zhizhuo@163.com (Z.S.); ysun@nmemc.org.cn (Y.S.)

**Keywords:** bacteria, protists, diversity patterns, community assembly, biogeographical pattern

## Abstract

As transitional ecosystems between land and sea, estuaries are characterized by a unique environment that supports complex and diverse microbial communities. A comprehensive analysis of microbial diversity and ecological processes at different trophic levels is crucial for understanding the ecological functions of estuarine ecosystems. In this study, we systematically analyzed the diversity patterns, community assembly, and environmental adaptability of bacterial and protist communities using high-throughput sequencing techniques. The results revealed a higher alpha diversity for the bacteria than for protists, and the beta diversity pattern was dominated by species turnover in both communities. In addition, the two community assemblages were shown to be dominated by deterministic and stochastic processes, respectively. Furthermore, our results emphasized the influence of the local species pool on microbial communities and the fact that, at larger scales, geographic factors played a more significant role than environmental factors in driving microbial community variation. The study also revealed differences in environmental adaptability among different microbial types. Bacteria exhibited strong adaptability to salinity, while protists demonstrated greater resilience to variations in dissolved oxygen, nitrate, and ammonium concentrations. These results suggested differences in environmental adaptation strategies among microorganisms at different trophic levels, with bacteria demonstrating a more pronounced environmental filtering effect.

## 1. Introduction

Estuaries, as transitional ecosystems between land and sea, play a crucial role in supporting a wide range of organisms, and thus biodiversity. Under the dual influence of natural factors and anthropogenic disturbances, estuaries have developed unique hydrological characteristics and environmental gradients [1]. The physical and chemical conditions in these ecosystems exhibit significant natural heterogeneity, shaped by the mixing of freshwater and seawater as well as by the transportation of dissolved substances and suspended particles [2]. Microorganisms, a key component of estuarine ecosystems, are highly sensitive to environmental changes. Drastic salinity fluctuations, particularly, significantly regulate the structure and function of microbial communities [3]. Furthermore, human activities, such as port construction, industrial development, and agricultural pollution, have exacerbated the nutrient load in estuarine ecosystems, further enhancing the spatial heterogeneity of microbial communities [4,5]. In this context, the composition, diversity, and assembly of estuarine microbial communities are subjected to significant dynamic changes. Therefore, a comprehensive analysis of the diversity patterns, biogeographical distribution, and community assembly mechanisms of these microorganisms is crucial for revealing the dynamic balance of estuarine ecosystems and their ability to cope with environmental changes.

The recent advancements in high-throughput DNA sequencing technology have enabled more thorough investigations into the spatial diversity patterns of microbial communities and associated driving mechanisms [6]. Earlier research has demonstrated that the assembly of microbial communities is influenced not only by the external environment, but also by the intrinsic characteristics of species [7]. Different types of organisms may exhibit different community assembly patterns within the same ecosystem [8]. For example, a study of the coastal and shelf ecosystems in the China Seas confirmed that the assembly of microeukaryotic communities is primarily determined by dispersal limitation, whereas that of prokaryotic communities is driven by homogenous selection. These differences largely stem from the intrinsic nature of microbial life, which necessitates the generation of vast biodiversity to sustain ecological functions [9]. Generally, organisms at higher trophic levels tend to exhibit lower biodiversity in specific regions compared with those at lower trophic levels [10,11], and less-biodiverse communities demonstrate greater vulnerability when facing environmental changes [12]. Furthermore, variations in biogeographical patterns may be due to differences in regional species pools at local scales [13]. Organisms of different types display slight variations in body size, dispersal capabilities, and metabolic activity, which can affect their distribution patterns and abundance across regions [14,15]. However, most studies tend to overlook the influence of diversity levels and local species pools on the biogeographical distribution of microorganisms at different trophic levels, especially in complex estuarine environments.

As indispensable microbial taxa in estuarine ecosystems, bacteria and protists drive key biogeochemical processes, including nutrient transformation, organic matter decomposition, and carbon and nitrogen cycling [16,17,18]. They represent two crucial components of the marine microbial food web and share many relatively comparable traits, such as small body size and short generation times [19]. The present study systematically and comparatively analyzed the differences in community composition, diversity, and assembly mechanisms, as well as the biogeographical patterns of bacterial and protists communities at different trophic levels within estuaries in the same coastal region, using high-throughput sequencing technology. By integrating the analyses of community diversity based on both unweighted and weighted Unifrac distances, this study revealed the influence of local species pools and species abundance distributions on microbial communities, as well as the differences in environmental adaptability among microbial types. These findings not only provide valuable insights into the ecological processes regulated by microorganisms in estuarine environments, but also help us understand how microorganisms respond to global environmental changes, offering a scientific basis for the management of estuaries worldwide.

## 2. Materials and Methods

### 2.1. Study Area and Sampling

Based on the geographical characteristics of the Liaodong Peninsula, a total of 12 representative estuaries were selected for sampling in August 2021. Each estuary is equipped with one sampling site. The sampling map can be found in other article [20]. To ensure the reliability and repeatability of the data, three replicate water samples were collected at each site, for a total of 36 samples. During each sampling, 3 L of water were collected from 0.5 m below the surface and placed into sterile bottles: 2 L would be used for DNA filtration and 1 L for the measurement of environmental parameters. The samples were vacuum filtered through sterile 0.22 μm membranes within 12 h of collection. The filtered membranes were then frozen at −20 °C and transported to the laboratory for storage at −80 °C. DNA was extracted within 24 h.

### 2.2. Measurement of Environmental Factors

Eight environmental parameters were measured at each sampling site. Temperature, pH, dissolved oxygen (DO), and salinity were measured directly on site using a YSI1001 (YSI Inc., Yellow Springs, OH, USA), while ammonium (NH_4_^+^), nitrite (NO_2_^−^), nitrate (NO_3_), and phosphate (PO_4_^3−^) concentrations were determined in the laboratory using 1 L samples collected within the previous 24 h at each station, following standard analytical methods [21].

### 2.3. DNA Extraction, PCR Amplification, and Sequencing

Total DNA for the sequencing of the 18S rRNA gene (for protists) and 16S rRNA gene (for bacteria) was extracted using the PowerWater DNA Isolation Kit (Qiagen, Redwood City, CA, USA) [22]. DNA concentration and integrity were evaluated using NanoDrop (Thermo Fisher Scientific, Carlsbad, CA, USA) and 2% agarose gel electrophoresis, respectively.

Specific primers were used to amplify the V3-V4 region of the 16S rRNA gene in bacteria and the V9 region of the 18S rRNA gene in protists. They were F341 (5′-CCTAYGGGRBGCASCAG-3′) and R805 (5′-GGACTACNNGGGTATCTAAT-3′) for the bacterial gene and 1380F (5′-CCCTGCCHTTTGTACACAC-3′) and 1510R (5′-CCTTCYGCAGGTTCACCTAC-3′) for the protist gene [23,24]. Detailed PCR amplification methods can be found in other literature [25]. After amplification, the PCR products were analyzed via 1.5% agarose gel electrophoresis, followed by purification, quantification, and equal pooling of the DNA. The pooled DNA was subjected to genomic sequencing on an Illumina NovaSeq 6000 platform (San Diego, CA, USA), and Illumina paired-end libraries were subsequently constructed.

### 2.4. Bioinformatic Data Processing

The raw sequences were subjected to quality control to remove low-quality reads (including those with an average Phred score < 20), ambiguous bases, primer mismatches, and sequences shorter than 150 bp. The retained high-quality reads were sorted into their respective samples, based on unique barcodes at the reverse primer ends. Then, the DADA2 algorithm in QIIME 2 (v. 2022.2) was used to cluster these reads into amplicon sequence variants (ASVs) [26]. Representative sequences for each 16S and 18S ASV were selected using the default method. Taxonomic assignment was then performed based on the SILVA 138 [27] and PR2 v4.12.0 [28] databases. The species information for each ASV at various taxonomic levels was obtained, and the composition of microbial communities at each level was then quantified across samples.

### 2.5. Statistical Analysis

All statistical analyses were conducted in R v. 4.2.2. Alpha diversity indexes, i.e., Chao1, Shannon, Pielou, and phylogenetic distance (PD), were calculated for the estuarine bacterial and protist communities, using the “vegan” package. The Wilcoxon rank-sum test was used to examine differences in alpha and beta diversity between the two communities. Betapart analysis was performed using the “betapart” package, which decomposes beta diversity distances into nestedness and turnover. The “GUniFrac” package was applied to evaluate variations in the composition of bacterial and protist communities, based on both unweighted and weighted Unifrac distances. To analyze the impact of regional species pools and species abundance distributions on microbial communities, the relationship between the number of shared species and beta diversity distance for any two samples was evaluated using linear regression. Since unweighted Unifrac distances only consider species composition, they can be used to determine the impact of the local species pool. In contrast, weighted Unifrac distances, which incorporate both species composition and relative abundance, can be used to assess the impact of species abundance distributions on community assembly [29]. Null models were applied to separately explore the mechanisms of assembly in bacterial and protist communities [30]. The beta-nearest taxon index (betaNTI) between pairs of samples was first calculated to differentiate the contributions of deterministic and stochastic processes to community assembly. A |betaNTI| value > 2 indicates that deterministic processes dominate community assembly, while a value ≤ 2 suggests a greater influence of stochastic processes. At the same time, the Raup–Crick (RC) metric was used to classify communities according to the influence of five ecological processes, i.e., homogeneous selection, heterogeneous selection, homogenizing dispersal, dispersal limitation, and drift. Euclidean distances and geographic distances between pairs of samples were calculated using the “vegan” and “geosphere” packages, respectively. Linear regression analyses for the comparison of bacterial and protist communities were conducted using both weighted and unweighted Unifrac distances [31]. Finally, variation partitioning analysis (VPA) was employed to assess the relative contributions of environmental and spatial factors to the formation of different communities. The correlation between environmental variables and microbial community composition was examined via Mantel tests performed using the “LinkET” package. The “TITAN2” and “picante” packages were used to calculate the environmental breadth of bacterial and protist communities in relation to the measured environmental variables [32] and to assess the phylogenetic signal [33], respectively. The values of these two parameters indicate the capacity of a community to adapt to a specific environmental factor, with higher values reflecting stronger adaptation [34].

## 3. Results

### 3.1. Differences in Alpha Diversity and Composition Between Bacterial and Protist Communities

**Figure 1:**
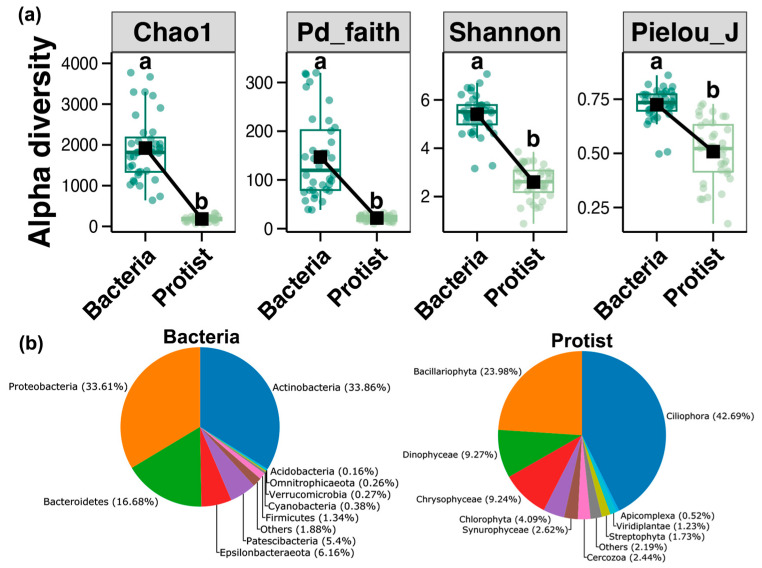
(**a**) Alpha diversity indexes for the bacterial and protist communities. Significant differences between groups are indicated by different letters (a, b). (Wilcoxon rank-sum test, *p* < 0.05). (**b**) Relative abundances of bacteria and protists at the phylum level.

The differences in alpha diversity among microorganisms at different trophic levels were determined by calculating the following four alpha diversity indexes: Chao1, Pd_faith, Shannon, and Pielou_J (Figure 1a). The results of differential analysis revealed significantly higher values for the bacterial community than for the protist community (Wilcoxon rank-sum test, *p* < 0.05). Actinobacteria (33.86%) and Proteobacteria (33.61%) were the dominant bacterial phyla, followed by Bacteroidetes (16.68%). In contrast, Ciliophora (42.69%) was the most abundant group in the protist community, followed by Bacillariophyta (23.98%), Dinophyceae (9.27%), and Chrysophyceae (9.24%), which comprised the next most-prevalent groups (Figure 1b).

### 3.2. Diversity Patterns of Bacterial and Protist Communities

**Figure 2:**
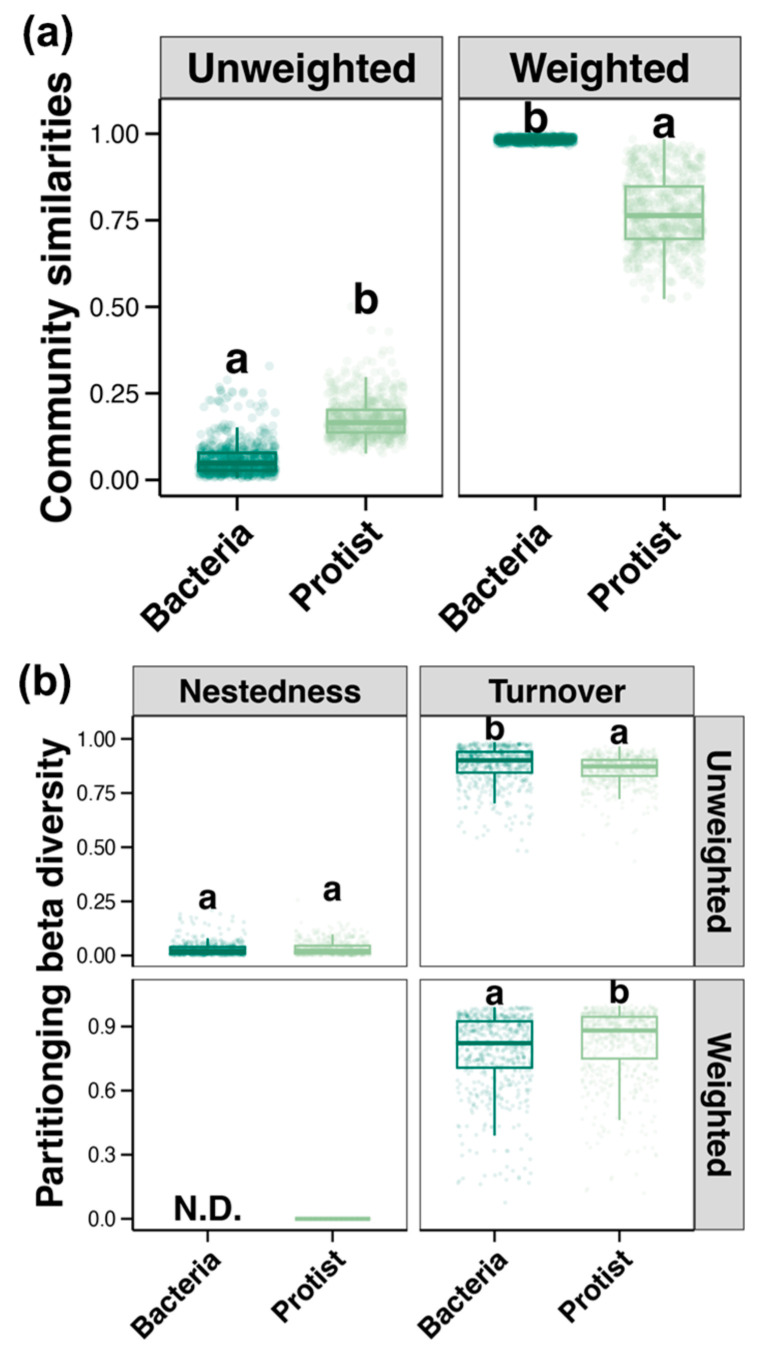
Beta diversity of bacterial and protist communities based on unweighted and weighted Unifrac distances (**a**) and differences in the turnover and nestedness of beta diversity patterns (**b**) (Wilcoxon rank-sum test, *p* < 0.05. Significant differences between groups are indicated by different letters “a” and “b”. “N.D.” indicates not detected).

Unweighted and weighted Unifrac distances were calculated separately for bacterial and protist communities. The unweighted Unifrac distances revealed that the beta diversity of protist communities varied more widely than that of bacterial communities, indicating a more significant variation in species composition for the former group than for the latter among samples (Figure 2a). In contrast, the weighted Unifrac distances showed a greater variability for bacterial communities than for protist communities, suggesting that the abundance of bacterial species fluctuated more than that of protists. This implied that, while the composition of bacterial species was relatively stable among samples, the differences in abundance distributions were pronounced (Figure 2a). In addition, to reveal the mechanism underlying the variation in microbial communities, the beta diversity distance was decomposed into two processes, nestedness and turnover, via Betapart analysis (Figure 2b). The results showed that turnover was the dominant mechanism driving variations in both bacterial and protist communities. The unweighted Unifrac distances also indicated that turnover had a greater impact on bacteria than on protists. However, when using the weighted Unifrac distances, the opposite was observed. No significant differences were detected in the contribution of nestedness between the two groups. These results suggest that, in bacterial communities, species turnover is more related to species presence or absence, while in protist communities it is associated not only with this parameter, but also with abundance distribution.

### 3.3. Role of Local Species Pools

**Figure 3:**
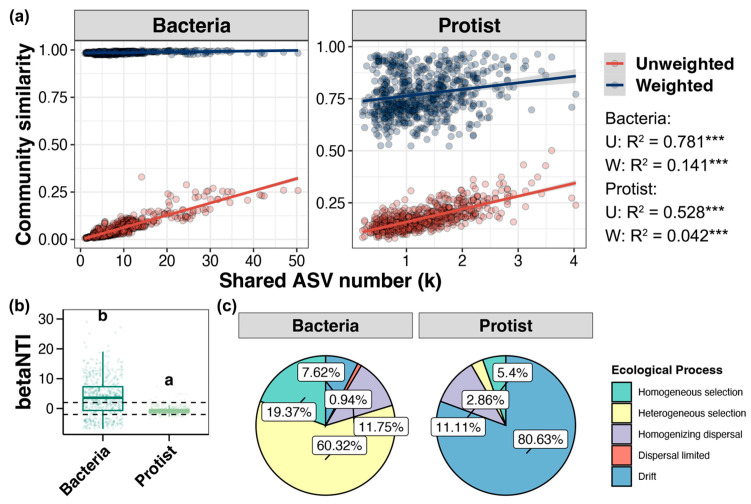
(**a**) Linear regression analysis of the number of shared ASVs between different samples and their corresponding beta diversity distance based on unweighted and weighted Unifrac distances. (**b**) Differences in the beta nearest taxon index (betaNTI) between bacterial and protist communities. Each point represents a pair of samples, with the dotted lines indicating the thresholds of 2 and −2, which separate deterministic processes from random processes. Significant differences between groups are indicated by different letters a and b. The symbol *** represents a significant correlation. (**c**) Relative importance of ecological processes in structuring the aggregation of bacterial and protist communities.

The correlation between the number of species shared by any two samples and their beta diversity distance was assessed by linear regression (Figure 3a). The results showed that the correlations based on unweighted Unifrac distances were significantly stronger than those based on weighted Unifrac distances for both bacteria and protists, indicating that the local species pool had a stronger influence on the microbial communities in the study area than relative species abundance. Furthermore, regardless of the use of unweighted or weighted Unifrac distances, the correlation between the number of shared species and beta diversity was stronger in bacterial communities than in protist communities, suggesting that the local species pool had a more pronounced effect on bacteria than on protists.

### 3.4. Mechanisms of Bacterial and Protist Community Assembly

The similarities and differences between the assembly mechanisms of bacterial and protist communities in the collected samples were analyzed by calculating betaNTI and RC distances, using a null model. The betaNIT values obtained showed that community assembly was mainly dominated by deterministic processes for bacteria and by stochastic processes for protists (Figure 3b). More specifically, bacterial assembly was dominated by the processes of heterogeneous selection (60.32%), homogeneous selection (19.37%), and homogenizing dispersal (11.75%), while protist assembly was mainly driven by drift (80.33%), with homogenizing dispersal (11.11%) being the second most important process (Figure 3c).

### 3.5. Relative Importance of Environmental and Geographical Factors

**Figure 4:**
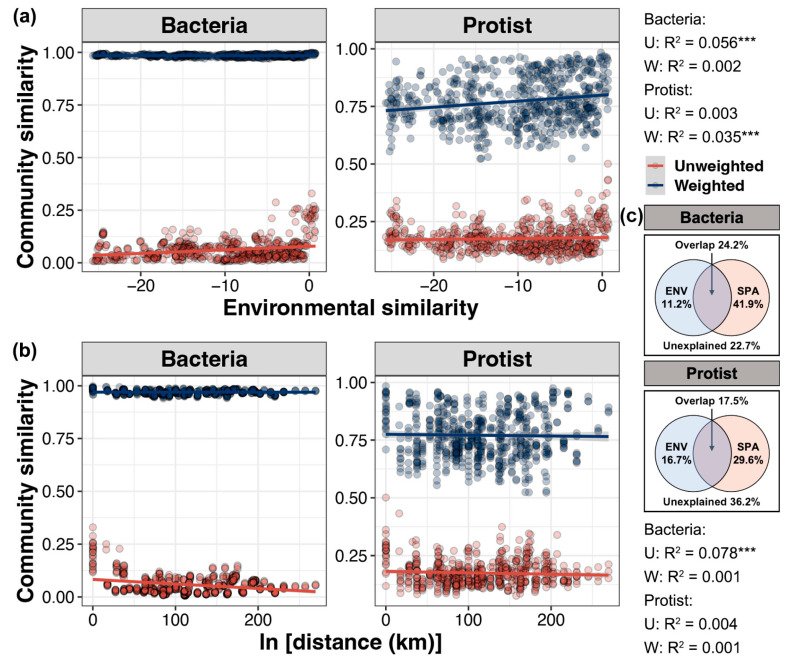
Linear regression of environmental similarity based on Euclidean distance (**a**) and geographic distance (**b**) with the similarities of bacterial and protist communities based on weighted and unweighted Unifrac distances. The symbol *** represents a significant correlation. (**c**) VPA analysis of bacterial and protist communities based on environmental variables and spatial factors.

The distance–decay pattern of similarity was analyzed to assess the relationship between environmental and geographic factors and the beta diversity of bacterial and protist communities (Figure 4a). The results showed that variations in environmental conditions were significantly correlated with the unweighted and weighted Unifrac distances of bacterial and protist communities, respectively (linear regression, *p* < 0.05). This finding suggests that environmental conditions primarily influence the presence or absence of bacteria, but affect both the survival and abundance of protists. The geographic distance between samples was significantly correlated only with the unweighted Unifrac distances of bacterial communities. For unweighted Unifrac distances, the distance-dependent similarity of geographic factors was higher than that of environmental factors, while for weighted Unifrac distances, environmental factors exhibited a stronger distance-dependent similarity than geographic factors. This result suggests that the impact of geographic factors on microbial communities is mainly reflected in the presence or absence of species (i.e., community composition), while environmental factors play a more significant role in the distribution of species abundance and changes in community structure. Additionally, VPA analysis further demonstrated that geographic factors were more important drivers of variation in bacterial and protist communities than environmental factors, with a greater influence on the former (41.9% vs. 29.6%) (Figure 4b).

### 3.6. Differences in Environmental Adaptation Between Bacteria and Protists

**Figure 5:**
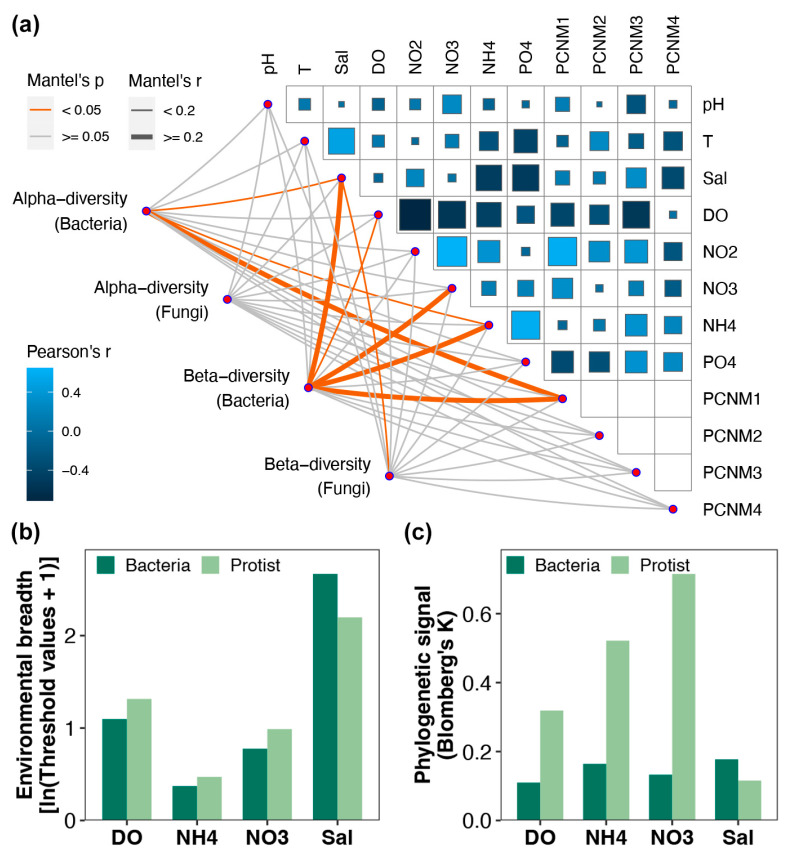
(**a**) Environmental drivers of alpha and beta diversity in bacterial and protist communities. The edge width and color correspond to the R value and statistical significance, respectively. The color gradient within the right-angle triangle indicates the Pearson’s correlation coefficient between environmental factors. Differences in environmental breadth (**b**) and phylogenetic signal (**c**) between bacterial and protist communities.

The Mantel test revealed that the alpha diversity of bacterial communities was significantly correlated with salinity, ammonium concentration, and the geographical factor PCNM1, while no significant correlations were found between the alpha diversity of protist communities and any environmental or geographical factors (Mantel test, *p* < 0.05, Figure 5a). The composition of bacterial communities was significantly correlated with salinity, DO, nitrate, and ammonium concentrations, and the geographical factor PCNM1, whereas that of protist communities was significantly correlated only with salinity. Additionally, the environmental breadth and phylogenetic signal for both bacteria and protists in response to salinity, DO, nitrate, and ammonium concentrations were calculated (Figure 5b,c). The bacterial communities exhibited a greater environmental breadth and stronger phylogenetic signal in response to salinity, indicating that they are more capable of adapting to changes in this parameter in estuarine regions compared to protists. In contrast, the protist communities demonstrated a greater environmental breadth and stronger phylogenetic signal in response to DO, nitrate, and ammonium concentrations, suggesting that they have a stronger capacity to withstand variations in these parameters.

## 4. Discussion

### 4.1. Differences in the Alpha Diversity and Composition of Bacterial and Protist Communities

Microbial alpha diversity indexes are important indicators that reflect the abundance of microorganisms and the characteristics of their community structure. In this study, four of these indexes (i.e., Chao1, Pd_faith, Shannon, and Pielou_J) were calculated, and the values for the bacterial communities in the estuaries of the Liaodong Peninsula were all shown to be significantly higher than those for the protist communities, which was consistent with expectations. Generally, organisms at high trophic levels have lower community diversity in a given area compared to organisms at low trophic levels [10,11]. Bacteria exhibit higher species diversity and abundance compared to protists, and their higher reproductive rates allow them to rapidly expand in different environments and maintain such high levels of diversity [35]. Furthermore, bacteria demonstrate strong environmental adaptability and resilience, with some of them (e.g., Bacilli) resisting adverse effects in harsh environments by producing dormant bodies [36]. This adaptability allows them to survive in different aquatic conditions, fostering a broad diversity. In contrast, protists (e.g., phytoplankton) have higher requirements for light and nutrients, which affects their ability to survive under unfavorable conditions and consequently limits their diversity [20].

This study revealed that Proteobacteria, Actinobacteria, and Bacteroidetes are widely distributed in the estuaries of Liaodong Peninsula, which is in line with the general bacterial distribution trends observed in numerous estuaries around the world [37,38]. Actinomycetes are usually widely distributed in freshwater and marine ecosystems and contribute to maintaining the stability of the local environment, especially where high concentrations of organic matter are present [38,39,40]. Additionally, Proteobacterioplankton and Bacteroidetes efficiently decompose dissolved organic matter consisting of molecular polymers, and play a key role in the cycling of carbon, nitrogen, and sulfur [41]. With regard to the protist community, our results showed Ciliophora (42.69%) as the dominant taxon, followed by Bacillariophyta (23.98%), Dinophyceae (9.27%), and Chrysophyceae (9.24%), which also exhibited relatively high abundances. This finding is consistent with previous studies of protist communities in temperate estuaries [42]. Ciliophora are widely distributed in these environments and are also an important component of marine microplankton communities. They prefer smaller prey, and serve as a key link in transferring energy from the microbial loop to higher trophic levels in the marine food web [43,44,45]. They are also considered as effective biological indicators of water quality and environmental pollution, due to their fast growth rate and responsiveness to environmental changes [46]. An important food source for Ciliophora is represented by Bacillariophyta. Certain species of this phylum, such as *Pseudovorticella coscinodisci*, form symbiotic relationships with specific Ciliophora spp. (e.g., *Coscinodiscus wailesii*) by attaching to their surface to assist in the accumulation of nutrients [47]. High abundances of Bacillariophyta may foster optimal conditions for the survival of Ciliophora.

### 4.2. Beta Diversity Patterns of Bacterial and Protist Communities Driven by Species Turnover

The results obtained based on unweighted and weighted Unifrac distances revealed a more significant variation in species composition among samples for protist communities than for bacterial communities, while the differences in species abundance were greater for the former than for the latter. Being smaller in size, bacteria have higher dispersal capabilities compared to protists, which results in a more even distribution [7]. Additionally, bacteria typically have broader niche widths, meaning that they exhibit greater metabolic plasticity compared to protists [48]. A single bacterial species can adapt to different environmental conditions by adjusting its abundance. As a result, the species composition of bacteria may not differ significantly between different samples, but their abundance fluctuates with environmental changes. The opposite is true for protists, whose narrower niche widths allow them only a relatively limited distribution across regions, causing their species composition to vary from place to place.

“Everything is everywhere, but the environment selects” is a concept widely accepted by many marine ecologists [49]. Our findings are consistent with this principle, showing that variations in bacterial and protist communities are primarily driven by turnover rather than nestedness, regardless of the application of unweighted or weighted Unifrac distances. Species replacement refers to the disappearance of certain species from an ecological community and their substitution with different ones. This turnover process may result from a variety of factors, including variations in habitat conditions, resource availability, climate, and disturbance, among others [50]. These factors may generate selective pressures in different areas, which in turn may contribute to changes in species composition [51,52]. In contrast, nestedness implies that organisms present in communities with lower species diversity can usually be found also in communities with higher species diversity. In other words, the distribution of species across different communities is characterized by a nested structure. This is more likely to occur in ecosystems with limited resources, more stable environments, or more adaptable species [53]. However, the contributions of turnover and nestedness to the dynamics of bacterial and protist communities vary depending on the metrics examined. In the present study, the unweighted Unifrac distances showed a stronger contribution of turnover for bacterial communities than for protist communities, while the weighted Unifrac distances showed the opposite. This suggests that in bacterial communities, turnover is more related to differences in species composition, whereas in protist communities it is associated with both species composition and abundance distribution. By combining the analyses of weighted and unweighted Unifrac distances, it was possible to achieve a more comprehensive understanding of the mechanisms underlying the variations in microbial community structure. Additionally, the results of this study also confirmed that bacteria and protists employ different strategies for environmental adaptation.

### 4.3. Deterministic Processes and Stochastic Processes, Respectively, Dominate the Assembly of Bacterial and Protist Communities

By assessing the correlation between the number of shared species in any two samples and their beta diversity distance, it was shown that the presence or absence of species (i.e., the local species pool) had a more significant impact on the assembly of regional microbial communities than species abundance. In addition, the contribution of the local species pool was shown to be greater for bacterial communities than for protist communities. These results imply that under environmental selection pressures, microbial distribution is not only dependent on species abundance, but is also influenced by whether species can adapt to and colonize specific environments. Maintaining the biodiversity of regional species pools is crucial for the sustainability of ecosystem functions and services at local or regional scales. Additionally, previous studies have shown that communities with low diversity levels are more vulnerable and sensitive to environmental changes [12]. Compared to bacteria, protists exhibit lower diversity; therefore, their niche selection is more constrained by habitat conditions or resource availability, due to the lower reliance on the local species pool.

Niche theory suggests that the composition of microbial communities is jointly influenced by deterministic and stochastic processes [54,55], whose relative importance depends on selection strength, dispersal rate, and the type and characteristics of microorganisms [56,57,58]. This study analyzed the similarities and differences in the mechanisms of bacterial and protist community assembly by calculating betaNTI and RC distances using a null model. The results showed that for these two groups, community assembly was predominantly driven by deterministic and stochastic processes, respectively. Organism size is considered a crucial factor influencing community aggregation [59]. The “size-dispersal” hypothesis predicts that smaller organisms have greater dispersal capabilities, and their distribution reflects the influence of environmental filters to a greater extent [60]. Our findings are consistent with this hypothesis, indicating that heterogeneous selection contributes more significantly to bacterial community assembly. Similar conclusions were reached in a study of lake microorganisms, showing that the community assembly of smaller and larger microorganisms was driven by deterministic and stochastic processes, respectively [61]. However, some reports have presented contrasting results, suggesting that smaller microorganisms are more influenced by stochastic processes at different ecological and geographical scales [62]. Therefore, further research covering a wider range of regions and ecosystems is needed to validate the general applicability of the findings obtained in our study.

In the present study, the bacterial community was shown to be dominated by heterogeneous selection (60.32%), which indicated a strong screening effect of different environmental conditions on these assemblages. The dominance of heterogeneous selection reflects the sensitivity of bacteria to environmental gradients, with differences in environmental conditions significantly affecting their composition and function. This dominance may also be due to the wide range of adaptations and metabolic diversity of bacteria, which can rapidly respond to environmental changes. The secondary roles of homogeneous selection (19.37%) and homogenizing dispersal (11.75%), on the other hand, suggest that some bacterial populations will exhibit similar ecological niche requirements and dispersal abilities in similar environments. This suggests that under some similar microhabitats, the bacterial community structure is more stable and has some dispersal capacity to maintain its similarity in neighboring areas. In contrast, the assembly of protist communities was shown to be mainly driven by drift (80.33%), which suggests that their population dynamics are more affected by stochastic processes and less responsive to environmental selection. This result may be related to the unique ecological characteristics of protists, which typically have larger body sizes and require higher nutrient levels, making them susceptible to the influences of food availability, predation pressure, and stochastic disturbances. Therefore, in regions with an uneven distribution of resources and unstable environmental conditions, the structure of protist communities is more affected by stochastic processes. At the same time, the observed contribution of homogenizing dispersal (11.11%) indicates that protists have a certain dispersal ability in specific regions, but this remains relatively limited, making it difficult for communities to adapt to complex environmental changes within a short period of time.

In summary, the differences described above reflect the distinct ecological strategies of bacteria and protists in terms of ecological adaptability, dispersal capacity, and environmental dependence. Our findings provide further evidence that the types or characteristics of organisms affect community dynamics and assembly [63].

### 4.4. Geographic Factors Are More Important Drivers of Variation in Bacterial and Protist Communities

The distance–decay pattern of community similarity is a common biogeographic indicator used to identify the potential drivers of community assembly [7]. According to this pattern, as geographic distance increases, the compositional similarity between any two sites declines [64]. In the present study, based on both unweighted and weighted Unifrac distances, the distance-dependent similarity of geographic factors was found to be higher than that of environmental factors when considering only the presence or absence of species. However, when species abundance was also taken into account, the opposite was observed. This result reflects how communities respond to different factors at different scales (geographic vs. environmental): at larger scales (geographic distance), differences in community composition are more pronounced, whereas at smaller scales, community abundance and structure are more driven by environmental factors. Furthermore, the results of VPA indicated that geographic factors were more important drivers of variations in bacterial and protist communities, with greater effects observed on the former. This finding does not conflict with the previously mentioned “size-dispersal” hypothesis. Although the smaller size of bacteria facilitates their dispersal over larger geographic areas, our study area spans various marine and estuarine regions, making geographic factors the primary drivers of variations in bacterial communities at larger scales.

### 4.5. Differences in Environmental Adaptability Between Bacteria and Protists

Abiotic environmental factors have been shown to directly or indirectly affect bacterial and protist communities in aquatic ecosystems. In this study, protist composition was significantly correlated with salinity, which is consistent with previous research [65]. At the same time, significant correlations were found between bacterial community composition and multiple environmental factors, such as salinity, DO, nitrate, and ammonium concentrations, demonstrating the high metabolic flexibility of these microorganisms. Bacteria can swiftly respond to environmental fluctuations, which makes them commonly used sensitive indicators of environmental changes. In addition, microorganisms at different trophic levels exhibit varying degrees of adaptability to environmental conditions [66,67]. Compared to protists, bacteria exhibit a greater environmental breadth and stronger phylogenetic signal in response to salinity in estuaries, suggesting that communities are more resilient to changes in this parameter. Estuaries, as transitional ecosystems where freshwater and saltwater meet, experience considerable salinity fluctuations [68]. Bacteria can cope with this stress through a variety of osmotic regulatory mechanisms (e.g., compatible solute accumulation, membrane adaptation, etc.) [69,70]. Some marine bacteria are even capable of surviving in freshwater environments [48]. In contrast, protist communities exhibit a greater environmental breadth and stronger phylogenetic signal in response to DO, nitrate, and ammonium concentrations, which indicates a stronger adaptability to changes in these factors. Protists typically function as primary or secondary consumers within ecosystems [71], where they directly or indirectly participate in nutrient cycling and exhibit strong adaptability to changes in nutrient gradients [72]. Overall, these findings highlight the different levels of resilience and adaptability of bacterial and protist communities to varying environmental factors, emphasizing the complex interactions between microorganisms and their habitat in aquatic ecosystems.

## 5. Conclusions

This study systematically revealed significant differences in community composition, diversity patterns, distance–decay relationships, assembly mechanisms, and environmental adaptability between temperate estuarine bacteria and protists. These differences reflect the distinct ecological functions and environmental response strategies of the two microbial communities. Bacteria exhibited higher alpha diversity levels and a greater ability to adapt to salinity changes, while protists demonstrated higher flexibility in terms of shifts in species composition and stronger responses to DO, nitrate, and ammonium concentrations. A common feature was that the beta diversity patterns of both bacterial and protist communities were primarily driven by turnover. By combining weighted and unweighted Unifrac distances, this study emphasized the key role of geographic factors in microbial community assembly and also highlighted the influence of the local species pool on regional microbial diversity. Furthermore, deterministic and stochastic processes were shown to dominate the assembly of bacterial and protist communities, respectively. These findings contribute to a deeper understanding of the ecology of microorganisms at different trophic levels in estuarine environments. However, given the limitations of our data, further research at larger temporal and spatial scales is needed to fully elucidate the impacts of geographic and environmental factors on microbial communities at different trophic levels.

## Data Availability

The datasets presented in this article are not readily available because the data are part of an ongoing study. For access to the dataset, please contact the corresponding author.
